# A meta-analysis comparing tenotomy or tenodesis for lesions of the long head of the biceps tendon with concomitant reparable rotator cuff tears

**DOI:** 10.1186/s13018-019-1429-x

**Published:** 2019-11-15

**Authors:** Yuyan Na, Yong Zhu, Yuting Shi, Yizhong Ren, Ting Zhang, Wanlin Liu, Changxu Han

**Affiliations:** 1 Department of Arthroscopy and Sports Medicine, The Second Hospital of Inner Mongolia Medical University, No. 1 Yingfang Street, Huimin District, Hohhot, 010000 Inner Mongolia Autonomous Region China; 2 Department of Spinal Surgery, the Second Hospital of Inner Mongolia Medical University, No. 1 Yingfang Street, Huimin District, Hohhot, 010000 Inner Mongolia Autonomous Region China; 3 Cardiac Function Department, Cadre Health Care Center, Inner Mongolia Autonomous Region People’s Hospital, Saihan District, Hohhot, 010000 Inner Mongolia Autonomous Region China; 4 Department of Pediatric Orthopedics, the Second Hospital of Inner Mongolia Medical University, No. 1 Yingfang Street, Huimin District, Hohhot, 010000 Inner Mongolia Autonomous Region China

**Keywords:** Biceps, Tenotomy, Tenodesis, Rotator cuff, Meta-analysis

## Abstract

**Background:**

The best treatment for lesions of the long head of the biceps tendon (LHBT) with concomitant reparable rotator cuff tears is still controversial. The purpose of the meta-analysis was to compare clinical outcomes of biceps tenotomy and tenodesis for LHBT lesions.

**Methods:**

A literature retrieval was conducted in MEDLINE, Embase, and Cochrane Library from 1979 to March 2018. Comparative studies (level of evidence I or II) comparing tenotomy and tenodesis for LHBT lesions with concomitant reparable rotator cuff tears were included. Risk of bias for all included studies was assessed using the Cochrane Collaboration’s risk of bias tool. Clinical outcomes compared were Popeye sign, Constant score, VAS pain score, cramping pain, elbow flexion and forearm supination strength, and re-tear of the rotator cuff.

**Results:**

Two randomized controlled trials (RCTs) and five prospective cohort studies (PCS) with 288 biceps tenotomy patients and 303 biceps tenodesis patients were included in this review. Tenotomy resulted in significantly greater rates of Popeye sign (RR, 2.70 [95% CI, 1.80 to 4.04]; *P* < 0.01) and a less favorable Constant score (MD, − 1.09 [95% CI, − 1.90 to − 0.28]; *P* < 0.01) compared to tenodesis. No significant heterogeneity was found between the two groups across all parameters except forearm supination strength.

**Conclusions:**

The current evidence indicates that biceps tenodesis for LHBT lesions with concomitant reparable rotator cuff tears results in decreased rate of Popeye sign and improved Constant score compared to biceps tenotomy.

**Trial registration:**

PROSPERO, CRD42018105504. Registered on 13 August 2018.

## Background

Lesions to the long head of the biceps tendon (LHBT), including traumatic injuries, inflammation, and subluxation, can cause significant shoulder pain and dysfunction. Conservative treatments for LHBT lesions consist of rest, ice compress, oral non-steroidal anti-inflammatory drugs, corticosteroid intra-articular injection, and physical therapy [1]. The current mainstream of surgical treatment for LHBT lesions is arthroscopic intervention.

Rotator cuff tears are commonly present in combination with LHBT lesions [2], possibly because these tears lead to greater pressure and friction on the biceps tendon [3]. LHBT lesions such as subluxation, dislocation, a biceps tear of more than 30%, or a degenerative superior labrum anterior to posterior type II lesion can result in chronic pain even after rotator cuff surgery [4–6], so treatment for these lesions during rotator cuff surgery is recommended [7–10]. Oliva et al. suggested two main treatments involving biceps tenotomy and tenodesis techniques which are recommended if symptoms persist for more than 3 months after conservative treatment [11].

During rotator cuff surgery, both tenotomy and tenodesis techniques are used to treat LHBT lesions, yet there is no consensus as to the superiority of either technique [12–15]. Tenotomy and tenodesis are the most common procedures to treat LHBT lesions and usually produce favorable clinical results. Biceps tenotomy is relatively quick, technically simple, improves symptoms, and allows for early rehabilitation [5, 8]. However, it can result in adverse outcomes such as loss of strength of elbow flexion and forearm supination as well as the cosmetic deformity known as the Popeye sign [16–20]. In contrast, biceps tenodesis can preserve the normal tension and power of the biceps muscle [21, 22], but it requires a technically more difficult procedure as well as a longer operation and rehabilitation [23, 24].

Several studies have reported the potential advantages of biceps tenodesis. A cohort study by Cho et al. suggested that both techniques show good outcomes including clinical and radiographic assessment, muscle strength, and range of motion, even though the incidence of Popeye deformity in the tenotomy group tended to be higher [25]. An RCT by Lee et al. found that the tenodesis group showed greater forearm supination power than the tenotomy group [26]. Similarly, a retrospective cohort study by Godenèche et al. found Constant scores to be significantly better for patients who had undergone tenodesis compared with those who had undergone tenotomy to treat LHBT lesions [27]. However, these advantages of tenodesis are not constant except for fewer incidences of Popeye deformity. Controversy persists as to whether tenotomy or tenodesis is preferable for treatment of LHBT lesions with concomitant reparable rotator cuff tears.

Several systematic reviews and meta-analyses have attempted to determine the superior technique for treatment of LHBT lesions with concomitant reparable rotator cuff tears [22, 28–30]. However, the conclusions drawn are based on results from nonrandomized controlled trials, which lack high quality evidence and therefore increase the likelihood of bias. We therefore sought to determine which technique is associated with (1) a lower risk of Popeye deformity, (2) a better Constant score, (3) lower visual analog scale (VAS) and cramping pain scores, (4) a lower risk of re-tear of the rotator cuff, and (5) better elbow flexion strength and forearm supination strength.

## Methods

### Searches

This study was designed and conducted according to PRISMA guidelines [31]. A literature retrieval was conducted in MEDLINE, Embase, and Cochrane Library from 1979 to March 2019. The following search words were used: (biceps tenotomy) AND (biceps tenodesis) AND (“rotator cuff” OR “supraspinatus” OR “supraspinatus tendon”). In addition, a manual search of the references of included studies was also performed to ensure no eligible studies were missed.

### Inclusion and exclusion criteria

The inclusion criteria were (1) comparative studies (level of evidence I or II) comparing tenotomy and tenodesis for LHBT lesions with concomitant reparable rotator cuff tears, (2) clinical outcomes assessed by clinical or functional scoring systems, (3) full text of studies available, and (4) published in English. The exclusion criteria were (1) not comparative studies and level of evidence I or II, (2) studies were not published in English, (3) biomechanical studies, and (4) animal studies.

### Data extraction

For each included study, two reviewers independently used a predefined form to extract data including first author, publication year, levels of evidence (LOE), sample size of each group, mean age of participants, gender distribution, and length of follow-up. The following clinical outcomes were also collected: Popeye sign, Constant score, VAS pain score, cramping pain, elbow flexion and forearm supination strength, and re-tear of the rotator cuff. The reviewers were not blinded to authors, journal of publication, or source of financial support.

### Assessment of risk of bias

The risk of bias was assessed for included studies using the Cochrane Collaboration risk of bias tool [32] which consists of the following items: random sequence generation, allocation concealment, blinding of patients and personnel, blinding of outcome assessment, incomplete outcome data, selective reporting, and other biases. Each included study was rated as having low, unclear, or high bias for each of the above items. Risks of bias figures were generated using Cochrane Review Manager 5.3 (Cochrane Collaboration, Nordic Cochrane Centre, Copenhagen, Denmark).

### Statistical analysis

Data analysis was performed via Cochrane Review Manager 5.3. Dichotomous variables (Popeye sign, cramping pain, re-tear of the rotator cuff) were presented as risk ratios (RRs) or risk difference with 95% confidence intervals (CIs). Continuous data (Constant score, VAS pain score, elbow flexion strength and forearm supination strength) were measured as mean differences (MD) with 95% CI. *P* < 0.05 was considered statistically significant.

Heterogeneity among included studies was assessed using the Q-statistic and *I*^2^ tests [33]. If *P* > 0.05 or *I*^2^ < 50%, the included studies were considered to have low heterogeneity and the fixed-effects model was applied to outcome data; otherwise, the random-effects model was applied.

## Results

### Study selection

**Fig. 1:**
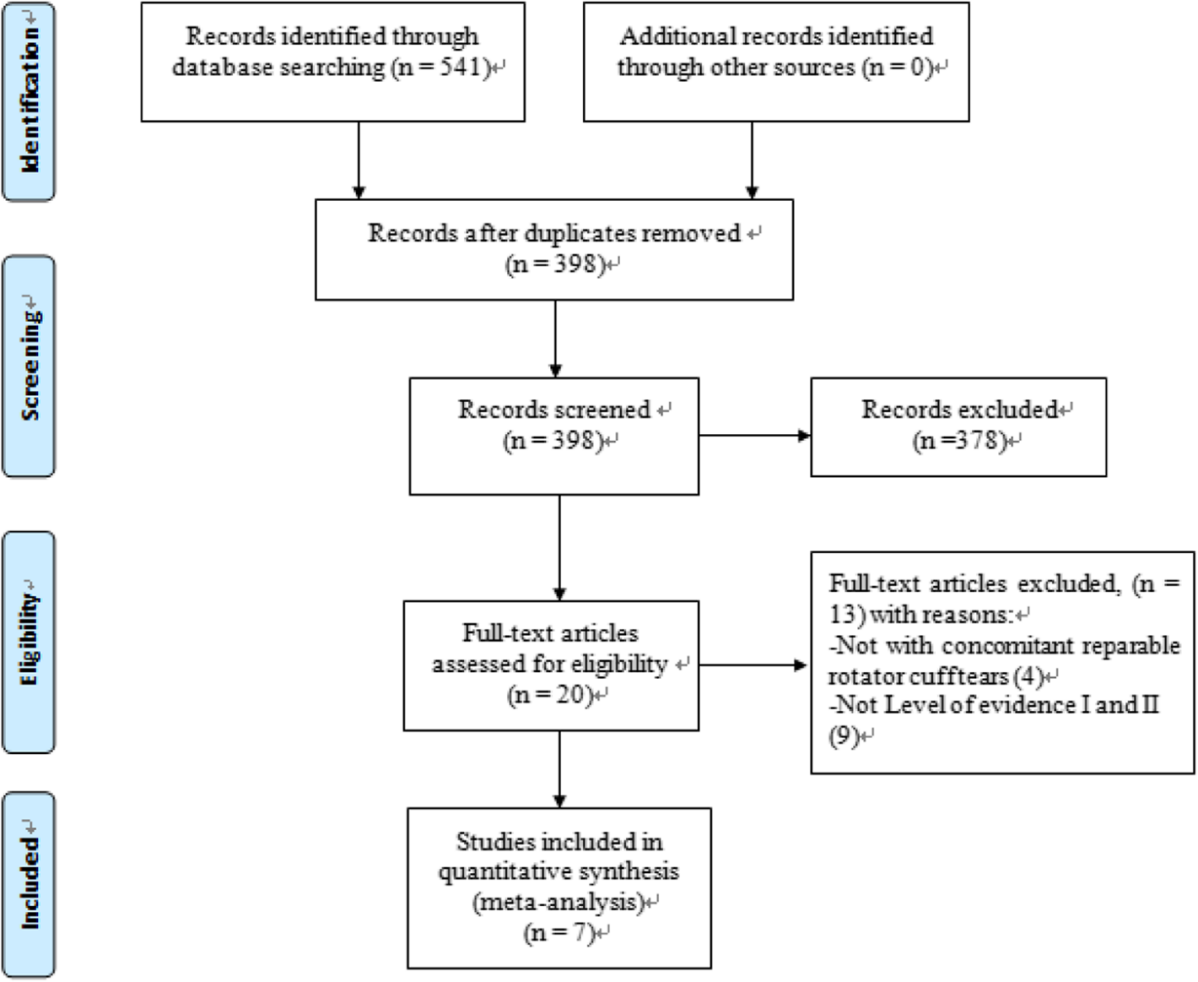
Flow diagram of study selection

The initial search of the online databases identified a total of 541 studies. After removing the duplicates, 398 publications were left. Screening the titles and abstracts excluded a further 378 studies based on predefined inclusion criteria. The full text of the remaining articles was then read and a further 13 articles were excluded. Eventually, 7 comparative studies with LOE I or II were included in this review (Fig. 1) [12, 13, 26, 34–37].

### Study characteristics

The main characteristics of the included studies are listed in Table 1. Two RCTs and five PCS with 288 biceps tenotomy patients and 303 biceps tenodesis patients were included in this review. The LHBT and rotator cuff injury type and tenodesis methods of the included studies are given in Table 2.

**Table 1:** Study characteristics and patient demographics of the included studies

Author (year)	Study, LOE	Sample size		Age, mean (range), year		Sex, M/F, *n*		Follow-up, months	
		Tt	Td	Tt	Td	Tt	Td	Tt	Td
Castricini (2018)	PCS, I	31	24	59.9 (40–71)	57.1 (40–70)	14/17	7/17	24.00	24.00
Lee (2016)	RCT, I	56	72	62.8 (55–77)	62.9 (50–75)	11/45	18/54	25.10	19.70
Oh (2016)	PCS, II	27	31	61.04 (53–69)	56.61 (42–76)	9/18	21/20	21.98	21.46
Zhang (2015)	RCT, I	77	74	61 (55–67)	61 (55–71)	36/41	35/39	25.00	25.00
Kukkonen (2013)	PCS, II	26	24	62.7(F); 63.7(M)	54.1(F); 54.9(M)	13/13	15/9	24.00	24.00
De Carli (2012)	PCS, II	30	35	59.6	56.3	NG	NG	23.00	25.00
Koh (2010)	PCS, II	41	43	66 (55–82)	65 (55–77)	9/32	16/27	27.93	27.05

*PCS* prospective cohort study, *RCT* randomized controlled trial, *LOE* levels of evidence, *Tt* tenotomy, *Td* tenodesis, *M* male, *F* female, *NG* not given

**Table 2:** LHBT injury types, rotator cuff injury types, and biceps tenodesis methods of the included studies

Author (year)	LHBT injury type	Rotator cuff injury type	Biceps tenodesis methods
Castricini (2018)	Tenosynovitis, subluxation, dislocation, or partial tear of the tendon	Grade I or II full-thickness reparable supraspinatus tendon tear	A interference screw
Lee (2016)	Partial tear	Small- to medium-sized rotator cuff tear	A interference screw
Oh (2016)	Partial tear	Full-thickness tears of the supraspinatus (and infraspinatus), high-grade partial-thickness supraspinatus tears, and full-thickness subscapularis tears with supraspinatus (and infraspinatus) tears	A suture anchor
Zhang (2015)	Severe inflammation, hypertrophy, instability, partial thickness tears, SLAP lesions	Small to large full-thickness rotator cuff tears	A suture anchor
Kukkonen (2013)	Irritated/frayed and/or unstable biceps tendon	Full-thickness supraspinatus tendon tear	Nonabsorbable titanium suture anchor
De Carli (2012)	Degenerative tears, tenosynovitis, subluxation, and SLAP lesions	Small to large rotator cuff tear	Suturing biceps tendon to cuff tendons
Koh (2010)	Tear more than 30%, subluxation or dislocation, or degenerative SLAP type II lesion	Rotator cuff tear	A suture anchor

*LHBT* long head of the biceps tendon, *SLAP* superior labrum from anterior to posterior

### Assessment of risk of bias

**Fig. 2:**
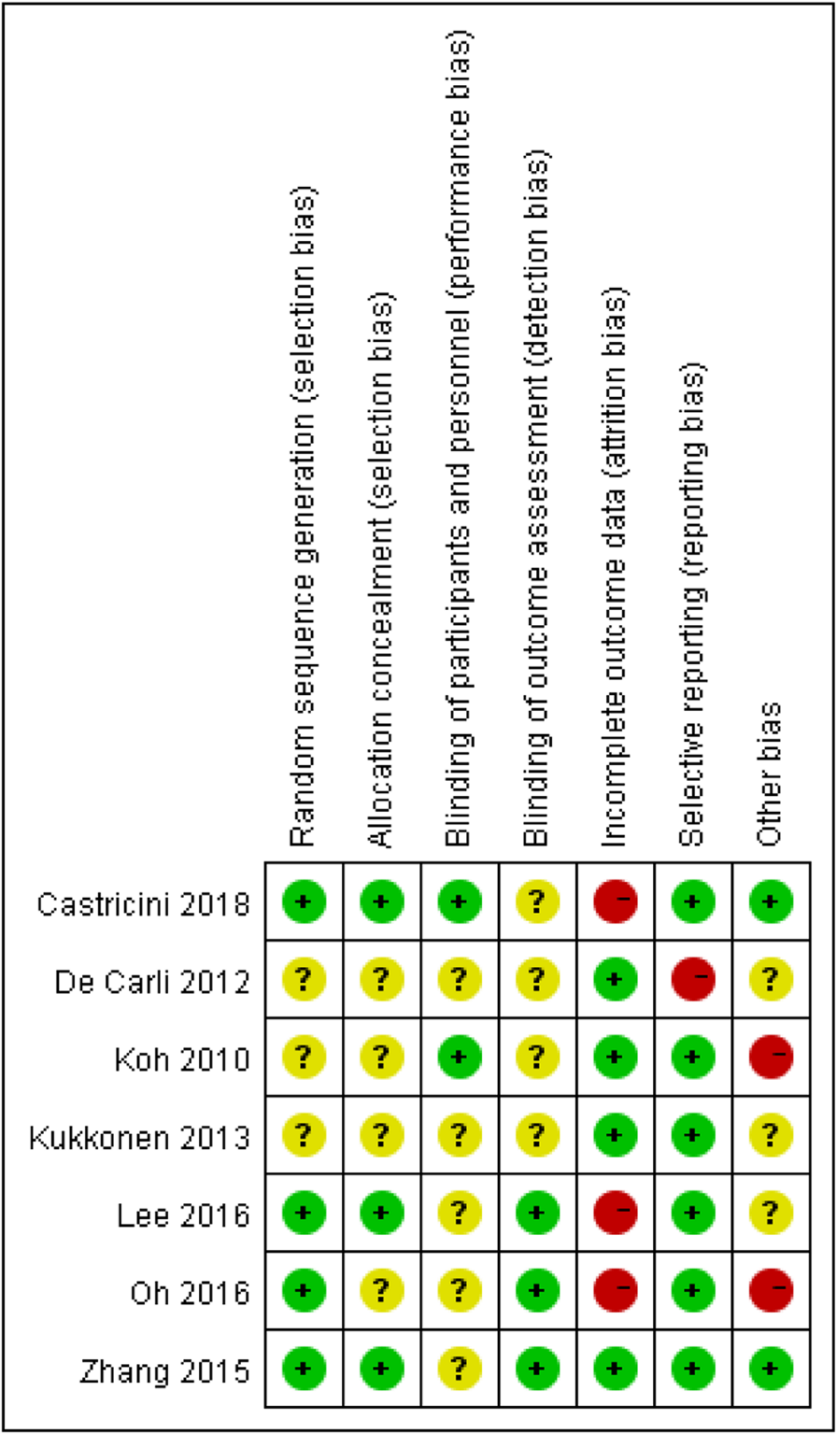
Risk of bias of included studies. +, low risk; −, high risk; ?, unknown risk

The risk of bias of the included studies is shown in Fig. 2. Two studies lost more than 20% of enrolled patients to the last follow-up and were therefore rated as having a high risk of attrition bias [36, 37]. The study by Lee et al. did not report the standard deviation of VAS pain score and standard deviation of Constant score at the last follow-up, which was thus judged as having a high risk of attrition bias as well [26]. The study by De Carli et al. only reported clinical outcomes of Popeye sign and Constant score, which was regarded as having a high risk of reporting bias [34]. Finally, patients with concomitant superior labrum-biceps complex lesions and rotator cuff tears were compared by Koh et al. and Oh et al., which may result in a high risk of potential other bias [12, 36].

### Synthesis of results

#### Popeye sign

**Fig. 3:**
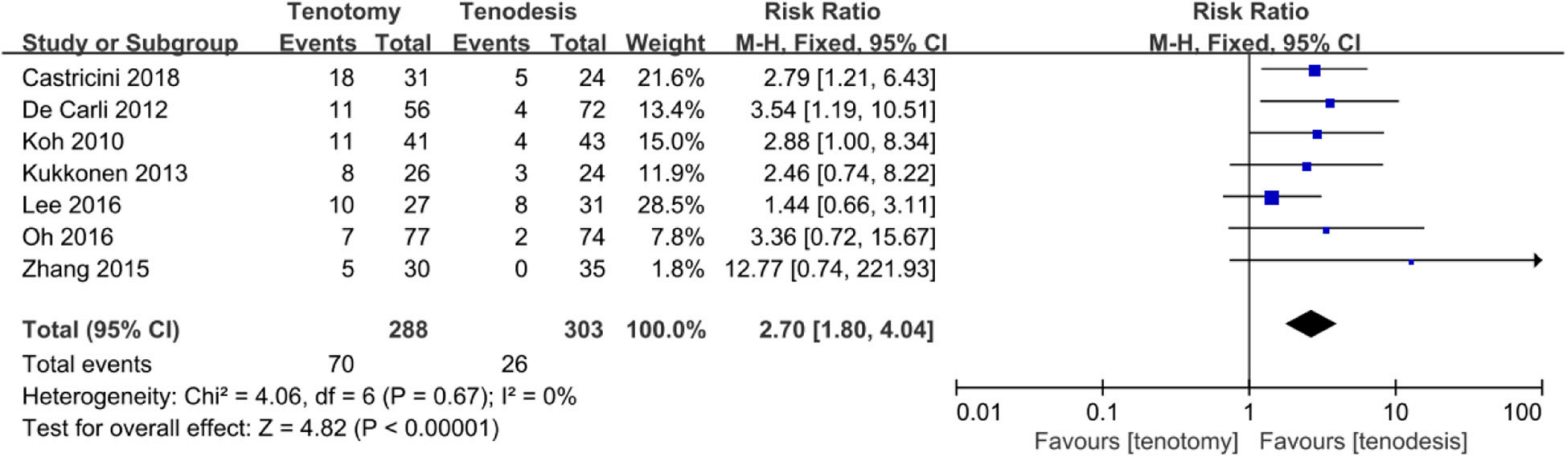
Forest plot diagram showing Popeye sign compared between biceps tenotomy and biceps tenodesis for LHBT lesions with concomitant reparable rotator cuff tears

Popeye sign was reported in all of the included studies. With biceps tenotomy, 24.3% of patients had Popeye sign, and with biceps tenodesis, 8.6% of patients had Popeye sign. There was a statistically significant difference in favor of the biceps tenodesis group (RR, 2.70 [95% CI, 1.80 to 4.04]; *P* < 0.01) compared with the tenotomy group. No significant heterogeneity was found between the two groups (*I*^2^ = 0%; *P* = 0.67, Fig. 3).

#### Cramping pain

Cramping pain was reported in 5 studies, with 202 patients treated with biceps tenotomy and 196 with biceps tenodesis. With biceps tenotomy, 7.9% of patients had cramping pain, and with biceps tenodesis, 5.6% of patients had cramping pain. There was no significant difference (RR, 1.35 [95% CI, 0.67 to 2.74]; *P* = 0.40) or heterogeneity (*I*^2^ = 0%; *P* = 0.41) between the two groups.

#### Constant score

**Fig. 4:**
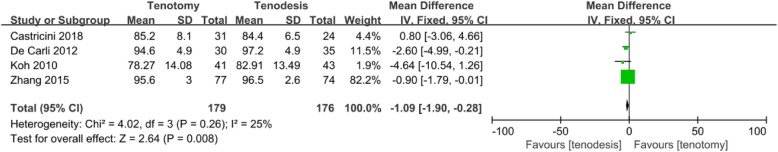
Forest plot diagram showing Constant score compared between biceps tenotomy and biceps tenodesis for LHBT lesions with concomitant reparable rotator cuff tears

Five studies reported Constant score, but of these, four studies could be used for meta-analysis as one study (Lee et al. [26]) did not report the standard deviation of Constant score and VAS pain score at the last follow-up. Across these studies, 179 patients were treated with tenotomy and 176 patients with tenodesis. There was a statistically significant difference in favor of the tenodesis group (MD, − 1.09 [95% CI, − 1.90 to − 0.28]; *P* < 0.01). No significant heterogeneity was found between the two groups (*I*^2^ = 25%; *P* = 0.26, Fig. 4).

#### VAS pain score

VAS pain score was analyzed in three studies. Across these, 135 patients were treated with tenotomy and 129 patients with tenodesis. There was no significant difference (MD, − 0.01 [95% CI, − 0.32 to 0.30]; *P* = 0.94) or significant heterogeneity between the two groups (*I*^2^ = 0%; *P* = 0.85).

#### Elbow flexion and forearm supination strength

Elbow flexion and forearm supination strength were reported in four studies including 201 patients treated with tenotomy and 220 patients with tenodesis. There was no significant difference between the two groups in terms of elbow flexion strength or heterogeneity (MD, 0.01 [95% CI, − 0.01 to 0.03]; *P* = 0.40; *I*^2^ = 0%); there was also no significant difference in terms of forearm supination strength although there was significant heterogeneity (MD, − 0.12 [95% CI, − 0.28 to 0.03]; *P* = 0.11; *I*^2^ = 95%).

#### Re-tear of the rotator cuff

Re-tear of the rotator cuff was found in three studies including 114 patients treated with tenotomy and 127 patients with tenodesis. There was no significant difference between the two groups (RR, 1.30 [95% CI, 0.66 to 2.56]; *P* = 0.46) and no significant heterogeneity (*I*^2^ = 0%; *P* = 0.50).

## Discussion

This meta-analysis was performed to compare the clinical outcomes of tenotomy and tenodesis for LHBT lesions with concomitant reparable rotator cuff tears. The most important findings from this review are that biceps tenodesis resulted in a higher Constant score and a lower risk of Popeye sign compared with biceps tenotomy. The results also revealed that there was no significant difference between the two groups in terms of VAS pain score, elbow flexion and forearm supination strength, risk of re-tear of the rotator cuff, or cramping pain.

Popeye sign is often seen as a drawback of biceps tenotomy [38]. Kelly et al. found 82.7% of men and 36.5% of women who undergoing biceps tenotomy had a positive Popeye sign [9]. Popeye sign frequently occurs in patients treated with tenotomy, and several meta-analyses have shown a significant difference with tenodesis. One prospective randomized trial also found that Popeye sign appeared more frequently in the tenotomy group than in the tenodesis group although this was not significant [35]. The occurrence of Popeye sign from the seven studies included in the current study is consistent with previous reviews. Although Popeye sign was relatively rare and was not a significant clinical outcome in patient satisfaction, the related cramping pain and fatigue were mentioned. Cramping pain was seen more frequently in the tenotomy group (7.9%) than in the tenodesis group (5.6%) in our study, but this difference was not significant; this is in contrast to results from Ge et al. and Gurnani et al. [29, 30].

The Constant score is the most widely used scoring instrument to assess shoulder function. A meta-analysis derived from RCTs and cohort studies concluded that there was no significant difference in Constant score between the two groups [30]. However, Shang et al. and Ge et al. reported that the tenodesis group had a significantly higher Constant score than the tenotomy group in their systematic review and meta-analysis [22, 29]. In the current study, the pooled Constant scores are consistent with the studies by Shang and Ge [22, 29]. Although none of the individual studies showed a significant difference in Constant score between the two procedures, the pooled result showed a significantly higher Constant score in the tenodesis group; this is a meaningful finding statistically.

No significant heterogeneity was found between the two groups except for forearm supination strength. This could be because of differences in measuring instrument, time of measurement, or tenodesis method. In terms of measurement technique, Lee et al. used a digital force gauge transducer [26], Oh et al. used a tensionometer [36], and Zhang et al. used a Chatillon digital dynamometer [35]). In terms of time of measurement, Lee et al. and Oh et al. took measurements at the last visit [26, 36] whereas this was not stated by Zhang et al. [35]. The heterogeneity could also be explained by different tenodesis methods. Scheibel et al. recommended bony fixation anchor tenodesis rather than soft tissue tenodesis because anchor fixation provides significant clinical and structural outcome advantages, such as better results in the LHBT score and in the evaluation of the structural integrity of LHBT [39]. Other characteristics, such as differences in surgical skills and in LHBT injury types, could also have contributed to the significant heterogeneity.

The advantage of our review is that we analyzed the available comparative studies with LOE I or II only, which could provide convincing results compared with previous reviews. Statistically significant difference in favor of biceps tenodesis was observed for Constant score (*P* < 0.01), which is controversial in the previous studies and reviews. Although Ge and Gurnani found lower incidences of cramping pain in patients managed with tenodesis, the present evidence showed no significant difference in this complication rate between the two groups.

There are several limitations to this meta-analysis. First, the potential benefit of biceps tenotomy is reduced surgical time [35], but this could not be analyzed in the current study as only two included studies reported this variable. Secondly, there is a multitude of confounding factors across the included studies that may affect the findings, including variable LHBT injury types, rotator cuff injury types, size of tear, involvement of subscap, biceps tenodesis methods, and postoperative rehabilitation protocols. Although we attempted to perform the subgroup analysis according to these confounding factors, the small number of studies makes this difficult. Thirdly, the small number of included studies and patients may mean that the study was underpowered. Finally, a cost-effectiveness analysis of biceps tenodesis and tenotomy would also contribute to the clinical decision process, which was not analyzed due to lack of relevant data.

## Conclusion

The current evidence indicates that biceps tenodesis for LHBT lesions with concomitant reparable rotator cuff tears results in better Popeye sign rates and Constant scores compared with biceps tenotomy. Given the limited number of RCTs included, this conclusion should be further validated by more high-quality RCTs.

## Data Availability

All data generated or analyzed during this study are included in this published article.

## Abbreviations

CIs: Confidence intervals
LHBT: Long head of the biceps tendon
LOE: Level of evidence
MD: Mean differences
PCS: Prospective cohort study
RCTs: Randomized controlled trials
RRs: Risk ratios
VAS: Visual analog scale pain score
